# The Limited Utility of Multiunit Data in Differentiating Neuronal Population Activity

**DOI:** 10.1371/journal.pone.0153154

**Published:** 2016-04-25

**Authors:** Corey J. Keller, Christopher Chen, Fred A. Lado, Kamran Khodakhah

**Affiliations:** 1 Department of Neuroscience, Albert Einstein College of Medicine, Bronx, NY, United States of America; 2 Department of Psychiatry and Behavioral Sciences, Stanford University School of Medicine, Stanford, CA, United States of America; 3 Stanford Neurosciences Institute, Stanford University, Stanford, CA, United States of America; 4 Department of Neurology, Montefiore Medical Center, Bronx, NY, United States of America; College de France, FRANCE

## Abstract

To date, single neuron recordings remain the gold standard for monitoring the activity of neuronal populations. Since obtaining single neuron recordings is not always possible, high frequency or ‘multiunit activity’ (MUA) is often used as a surrogate. Although MUA recordings allow one to monitor the activity of a large number of neurons, they do not allow identification of *specific* neuronal subtypes, the knowledge of which is often critical for understanding electrophysiological processes. Here, we explored whether prior knowledge of the single unit waveform of specific neuron types is sufficient to permit the use of MUA to monitor and distinguish differential activity of individual neuron types. We used an experimental and modeling approach to determine if components of the MUA can monitor medium spiny neurons (MSNs) and fast-spiking interneurons (FSIs) in the mouse dorsal striatum. We demonstrate that when well-isolated spikes are recorded, the MUA at frequencies greater than 100Hz is correlated with single unit spiking, highly dependent on the waveform of each neuron type, and accurately reflects the timing and spectral signature of each neuron. However, in the *absence* of well-isolated spikes (the norm in most MUA recordings), the MUA did not typically contain sufficient information to permit accurate prediction of the respective population activity of MSNs and FSIs. Thus, even under ideal conditions for the MUA to reliably predict the moment-to-moment activity of specific local neuronal ensembles, knowledge of the spike waveform of the underlying neuronal populations is necessary, but not sufficient.

## Introduction

The ability to simultaneously monitor the activity of multiple neuronal populations is of critical importance. Noninvasive techniques such as recordings from scalp EEG electrodes provide an overview of neuronal activity but fail to identify specific types of neurons, but more invasive approaches using microelectrodes can provide additional information. Microelectrode recordings can parse signals into low frequency activity (<250Hz), termed the ‘local field potential’ (LFP), and higher frequency activity (>250Hz), termed ‘multiunit activity’ (MUA). The LFP is thought to represent the “summed” synchronous excitatory and inhibitory post-synaptic events, whereas the MUA is thought to result from the action potential firing of a combination of neuronal subtypes.

MUA can sometimes be analyzed further to isolate the activity of single neurons whose spiking provide a fundamental measure of brain function. However, there are many situations when microelectrode recordings do not easily permit isolation of the activity of single neurons. For example, gathering MUA without information from well-isolated spikes is common when chronic microelectrode recordings are performed in non-human primates [1–5] or in patients with tetraplegia [6, 7]. Even when single neuron recordings are feasible, oftentimes information about the population as a whole cannot be generalized from the recording of one or a few neurons.

In the situations described above—which comprise a large fraction of animal and human electrophysiological experiments—high frequency recordings are easily accessible and reflect neuronal spiking activity from distances on the order of 100μm [8, 9]. Recent studies have demonstrated that neuronal firing contributes to frequencies as low as 100Hz and the power in the 100-200Hz range has been shown to correlate with spiking activity in human [10] and rodent hippocampus [11, 12] as well as in non-human primate visual cortex [13, 14]. Clearly, if one could estimate the population activity of *specific neuronal subtypes* by using components of high frequency recordings it would be a major step forward and would greatly enhance our ability to monitor and scrutinize physiological processes. However, the contribution of spiking activity from neuronal subtypes to specific frequency bands is not well understood.

Two recent findings suggest that high frequency activity may be separable into frequency bands specific to neuronal populations. First, removing hippocampal pyramidal cell spikes (pyramidal neuron ‘despiking’) from high gamma (90-150Hz) recordings caused a larger decrease in power than interneuron despiking, suggesting that pyramidal cells contribute more than interneurons to activity in this frequency range [11]. Second, using modeling in the rat CA1 it is suggested that action potentials from basket cells contribute less to power in the high gamma range than do pyramidal neurons [15].

Based on these findings, we sought to test the hypothesis that different frequency bands within the MUA represent activity from specific populations of neurons. We evaluated whether microelectrode MUA data represents the spiking activity of neurons, and more specifically whether such data can allow one to infer differential activity of specific neuron types. To do so, we recorded spiking and high frequency activity in the MUA range in the dorsal striatum of awake, freely moving mice. We obtained average spike waveforms of two different neuronal types (medium spiny neurons—MSNs-, and fast spiking interneurons—FSIs) from the single unit data and used it to define specific frequency bands for analysis in the MUA. We then analyzed these frequency bands in simulated data in order to evaluate the utility of these frequency bands in predicting the population activity of specific neurons. We demonstrate that for a simulated population of neurons low frequency MUA correlated with the MSN spiking, while high frequency MUA correlated with the spiking activity of both MSNs and FSIs. Activity in low frequency MUA predicted the MSN population firing rate with an accuracy >70%, regardless of FSI activity. However, the overall accuracy of predicting the firing rate of *both* populations, even under these ideal conditions where the single unit waveform of the neurons was known, did not exceed 50%. Together, our study suggests that knowledge of underlying populations is critical to utilize high frequency activity to predict local ensemble dynamics, but not always sufficient.

## Results

Experiments were performed on 8–10 week old C57/Bl6 mice (N = 17). Spikes that did not meet the inclusion criteria (see Methods) were removed from the analysis. The remaining cells with well-isolated spikes (n = 138, N = 8 mice) were further categorized as MSNs (n = 43), FSIs (n = 25), or not meeting the criteria for either subtype (n = 70).

### Relationship between spiking and high frequency activity

**Fig 1:**
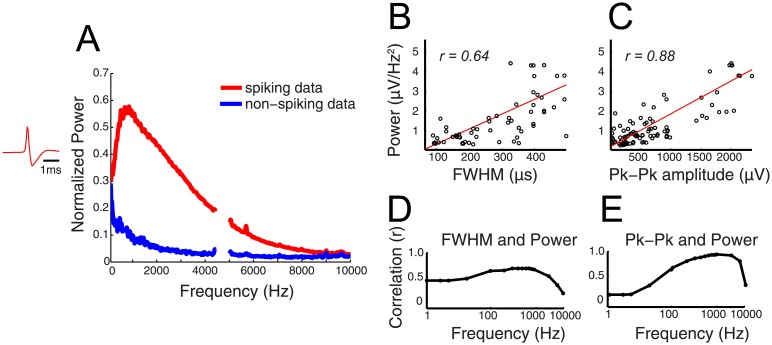
High frequency activity contains power from spikes above 100Hz. A) Frequency representation of data containing spikes and not containing spikes. The spike used to calculate the frequency decomposition is shown on the left. Power due to noise in the recording at 5KHz was removed from the analysis. B-C) Relationship of B) width and C) peak-peak amplitude of spikes (n = 70) to power in the 300-400Hz band. D-E) Comparison of spiking characteristics to power across frequency bands. The correlation value from B and C is calculated for each band and plotted in D and E, respectively. Time scale = 1ms. FWHM = full width at half maximum.

We first examined how action potentials are represented in the frequency domain by performing spectral analysis on isolated spikes. An increase in power from 1 Hz– 10 kHz was observed with peak increases from 200Hz - 1kHz during spiking time periods compared to non-spiking time periods (Fig 1A). Power in a representative frequency band (300–400 Hz) during an isolated spike exhibited a significant correlation with the width of the action potential (FWHM; Fig 1B; *r = 0.64; n = 138 cells*, *p < 0.01*, *Student’s t-statistic*, *df = 136*) and the peak-peak amplitude (Fig 1C; *r* = *.88; p <* *.01*, *df = 136*). Across all frequencies, the relationship of spectral power and both spike width and peak-peak amplitude was higher between 100–2000 Hz and decreased at the frequency range <100 Hz and >2 kHz (*r > 0.5*; Fig 1D and 1E).

Examining the effect of spiking characteristics (peak-peak amplitude, firing rate) on the frequency content of a spike waveform is difficult as spiking characteristics recorded extracellularly are not independent (e.g., in our dataset, action potentials with larger spike width tended to have lower firing rates). To remove this confound and examine the effect of one spiking attribute on frequency content, we selected a spike at random and modified either its amplitude or firing rate while constraining the other two variables. Increasing the firing rate (while constraining amplitude and spike width) or amplitude (while constraining firing rate and spike width) resulted in a strong correspondence between spiking characteristics and power in frequency bands above 50 Hz (*r*_*firing rate*, *power*_ *> .5 for frequencies 50-100Hz and > .9 for frequencies >100Hz; r*_*amplitude*, *power*_ *> .9 for frequencies >50Hz*; S1 Fig).

**Fig 2:**
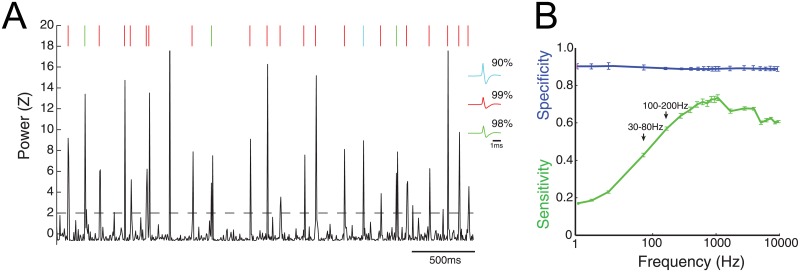
High frequency power can reliably detect spikes. A) 5s trace of power in 300-400Hz. Raster plots represent spikes whose color matches the waveform on left. Using a detection threshold (z > 2, dotted line), the sensitivity for three spikes on left was calculated. These percentages are shown next to their spike waveform. B) Sensitivity and specificity as a function of frequency for all cells, MSNs, and FSIs. Error bars denote SE. Time scale = 1 ms.

Because spiking attributes correlated with power in high frequencies, we hypothesized that increases in high frequency activity would reflect the timing of single spikes. For each neuron, we calculated the probability that high frequency activity increased during times of spiking. An example of this analysis using three simultaneously recorded spikes is found in Fig 2A. Power in a representative frequency band (300-400Hz) increased during time periods of spiking for these neurons (Fig 2A; *sensitivity*_*300-400Hz*_ = *0.90–0.99*). Group analysis of all neurons across all frequency bands demonstrated that high frequency power above 100 Hz detected spikes with a sensitivity of 55–73% and specificity 88–91% (Fig 2B).

### Neuronal cell types have specific high frequency signatures

**Fig 3:**
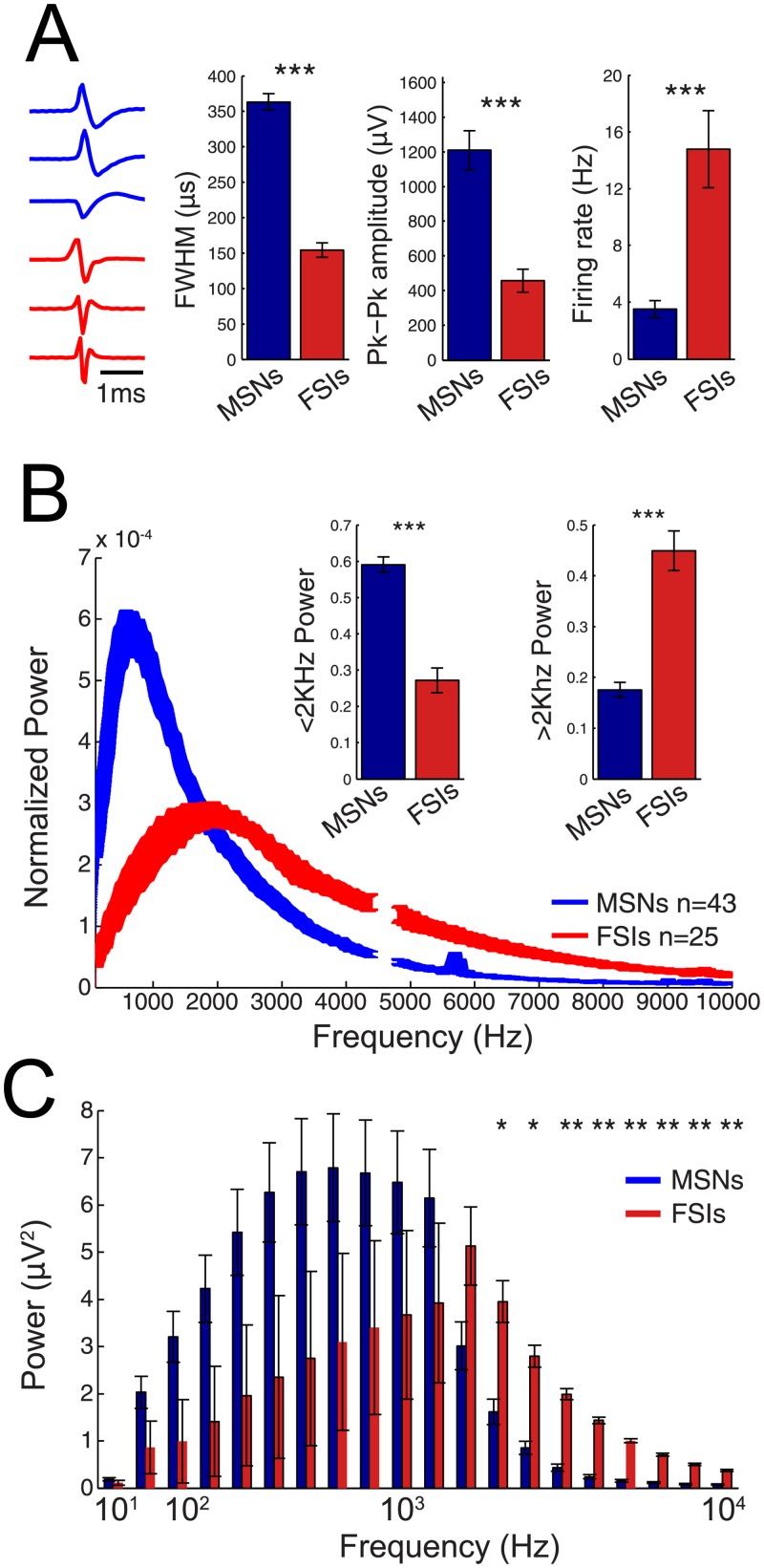
Neuronal cell types have specific high frequency signatures. A) *Left*: Example spike waveforms of MSNs and FSIs. *Right*: MSNs exhibit wider width, higher amplitude, and lower firing rates. B) Mean power spectrum of MSN and FSIs. Mesh represents standard error. Each spike is normalized by the area under the curve prior to averaging. *Insert*: Comparison of power above and below 2KHz. C) Total power contribution from well-isolated spikes in a given frequency band. Analysis takes into consideration power in each frequency band due to a single spike as well as the firing rate of that cell. Error bars denote SE. Time scale = 1ms. ***p < .001, **p < .01, *p < .05.

Next, we investigated if neuronal subtypes exhibit ‘spectral signatures’–that is, differential power distributions that may help identify time periods of spiking activity from specific populations. Previous analysis demonstrated that spiking characteristics (amplitude and spike-width) predict the power in high frequency bands (Fig 1). To this end, we next describe the spectral signatures in defined populations of cells to examine which frequency bands better represent each population. We first sorted spikes into medium spiny neurons and fast-spiking interneurons based on their spike-width and firing rate (see Methods for spike sorting procedure). The summary of the Spike waveform characteristics of the sorted cell populations are shown in Fig 3. As expected from our sorting definition for assigning cell type identity, MSNs had broader (larger FWHM) action potentials and lower firing rates, while FSIs on average had narrow action potentials and higher firing rates (Fig 3B, n_MSN_ = 43; n_FSI_ = 25; *p < .001*, *unpaired t-test*, *df = 24*), consistent with previous studies [16]. In addition, we found that once sorted to these cell types MSNs had larger amplitudes. This was likely due to the fact that during experiments for practical reasons faster spiking or large amplitude neurons were more likely to be selected for recordings. To remove this potential confound, the frequency decomposition of all spikes was normalized. Overall, compared to FSIs, MSNs contributed a significantly larger proportion of power in lower frequencies (< 2 KHz) and a smaller proportion of power in higher frequencies (> 2 KHz) (Fig 3B, *p < 0.0001*, *unpaired t-test*, *df = 24*).

As this analysis focused on the frequency content of single action potentials, to examine the power contribution of each cell in a recording segment, we adjusted for differences in firing rates by multiplying the normalized power of the spike waveform by the mean firing rate of each neuron. Across all recordings, a significant increase in overall power was observed from 100–1000 Hz in MSNs compared to FSIs (*p<0.05*, *unpaired t-test*, *uncorrected*, *df = 24*), but became non-significant when correcting for multiple comparisons (Fig 3C). On the other hand, FSIs exhibited a higher power contribution in frequencies >2 KHz when compared to MSNs. (Fig 3C, *p<0.01*, *unpaired t-test*, *df = 24*, *Bonferroni correction for multiple comparisons*).

**Fig 4:**
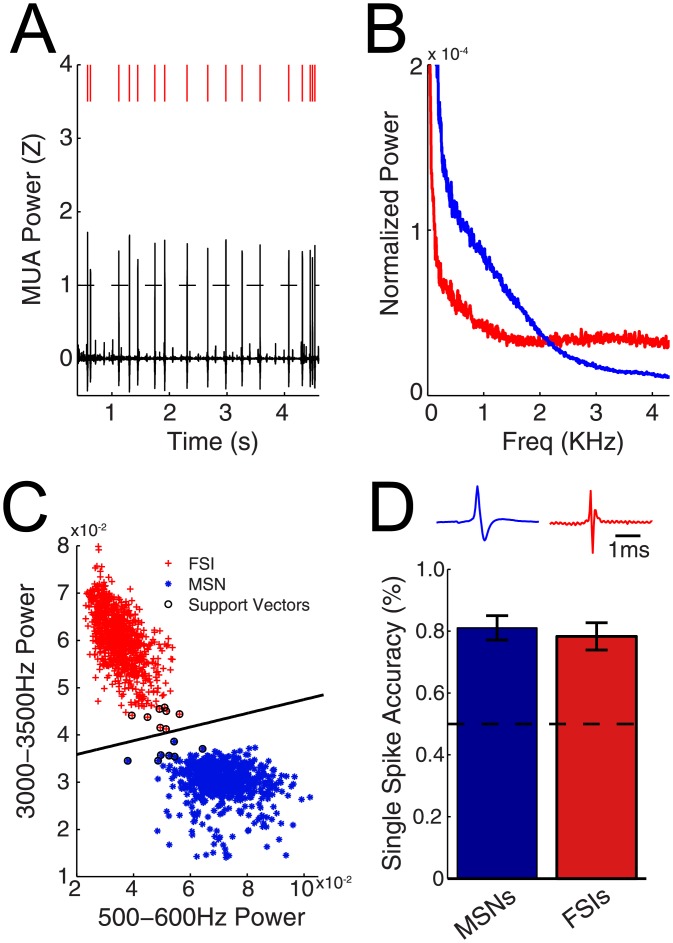
High frequency power can predict neuronal cell type from single spikes. A) BLP trace of recording. Raster plot represents times of increased activity above threshold (dotted line). B) Single trial power spectrum from time periods exhibiting high BLP. Features used for the binary classifier were extracted from the power spectrum as high and low power in frequencies. C) Support vector machine training set. Two channels were used for the training set. D) *Top panel*: Mean waveform traces extracted from time periods where the ratio of power in the high (3000-3500Hz) to low (500-600Hz) frequency bands were increased (red trace) and decreased (blue trace) as classified by the method illustrated in (C). *Bottom panel*: Group accuracy of classifying neuronal cell types based on spectral signature on a single trial basis. Power in low and high frequency bands were used as features in the binary classifier.

We then examined how well can one *detect and classify* spikes as MSNs or FSIs based on these spectral signatures. First, time points of multiunit power (>300 Hz) exceeding a set threshold were extracted (Fig 4A, black trace). During these times, spikes were often observed (Fig 4A, red lines). Next, wideband data during these periods were extracted and frequency decomposition was performed at the single-trial level. The mean frequency representation of putative spikes for two different recordings is illustrated in Fig 4B. For each putative spike, power in lower (500–600 Hz) and higher (3–3.5 KHz) frequency bands was used as features to classify the spike as either an MSN or FSI using a binary classifier (Fig 4C). These frequency bands were chosen because as discussed earlier they exhibited the strongest difference of power between the two cell types. The mean spike waveform of putative MSN and FSIs from this classification are shown in Fig 4D (top panel). Across all neurons, spikes from MSNs were correctly identified from the spectral power classifier with an accuracy of 80%, while spikes from FSIs were correctly identified with an accuracy of 78% (Fig 4D, bottom panel). Thus clearly, the classifier performed well above chance level when identifying both classes of neurons (dotted black line).

### High frequency activity is a poor predictor of ensemble dynamics

**Fig 5:**
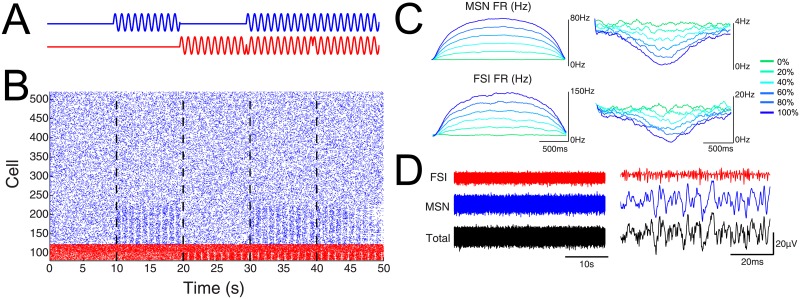
Modeling the spiking contribution to high frequency activity. A) Experimental design. Blue and red traces represent the times at which the firing rate of MSNs and FSIs were modulated, respectively. B) Raster plots of a subset of MSNs (blue) and FSIs (red) used in the simulation. In this example, 20% of neurons were modulated. C) Instantaneous population firing rates for MSNs and FSIs when different proportions of neurons were modulated. The left shows population firing rates as a function of the proportion of neurons modulated when the sinusoidal input is positive (so that the input increases firing rates). The right panel shows population firing rates when the input is negative (so that the input decreases firing rates). Traces are color-coded based on the proportion of neurons that were driven by the input. D) Aggregate high frequency activity derived from the total spiking of all neurons used in the model. FSI and MSN contributions as well as the total activity for the duration (left) and a subset (right) of the recording.

Following identification of a spectral signature for each neuronal subtype, we hypothesized that in the absence of a well-isolated spike populations of spikes will also exhibit spectral signatures that may be used to differentiate the properties of population activity on a moment-to-moment basis. To examine if high frequency activity ‘tracks’ population activity dynamics, we simulated resting and evoked responses from a population of cells with distributions and spiking attributes similar to the experimental data (see Methods for details). Evoked data consisted of times where MSNs, FSIs, or both populations were modulated. The proportion of the population that was responsive to the ‘evoked stimulation’ was also varied (0–100% of neurons). The simulation paradigm, raster plot of a subset of cells, instantaneous population firing rates of MSNs and FSIs during periods of increased and decreased evoked activity, and summed action potentials for MSNs and FSIs are shown (Fig 5A–5D). The details of the simulations are described in the methods section but, in brief, a sine wave was used to modulate the firing rate of MSN and FSI neurons at various time intervals to produce the following conditions: spontaneous firing of both MSNs and FSIs (no modulation; 0–10 s); MSN modulation (10–20 s), FSI modulation (20–30 s), modulation of MSNs and FSIs such that changes are negatively correlated (30–40 s), and modulation of MSNs and FSIs such that changes are simultaneous and positively correlated (40–50 s). Maximum instantaneous firing rates when 0% of neurons were modulated were 3.7 Hz for MSNs and 19.2 Hz for FSIs. At 100% modulation, maximum instantaneous firing rates increased to 78.2 Hz for MSNs and 141.5 Hz for FSIs. To test as many different conditions as possible, each simulation tested a wide range of population firing rates. For example, when 100% of MSNs were modulated, the population firing rates varied from 0 to 78.2Hz (Fig 5C). Finally, the aggregate signal based on the total spiking of all MSNs and FSIs used in the model was calculated. The total duration (left) and a subset (right) of these signals can be found in Fig 5D. The aggregate MSN and FSI signal was the signal which was used in subsequent analyses.

**Fig 6:**
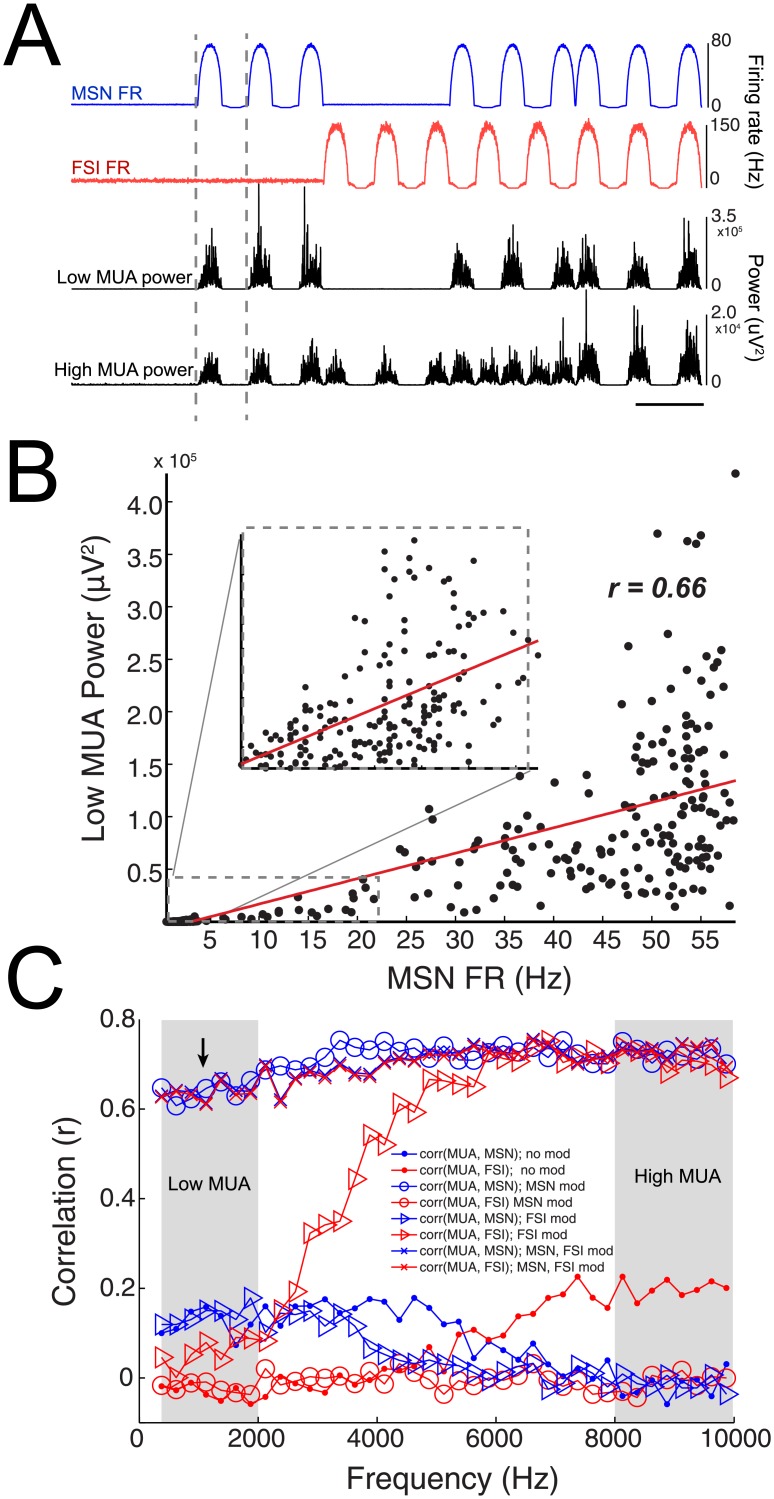
Low MUA tracks MSN spiking while high MUA tracks MSN and FSI spiking. A) Top: Traces of the firing rates (FR) of MSNs and FSIs. Bottom: power changes in low (0.3-2KHz) and high (8-10KHz) frequencies within the MUA range. B) Correlation between low MUA power and MSN firing rates for all firing rates in one cycle of modulation (gray dotted bars in A). Correlation for low MSN firing rates are expanded in the right panel. Correlation value from one cycle is denoted by the black arrow pointing to the blue circle in C. C) Correlation between power in different frequency bands and population MSN and FSI firing rates during spiking with no modulation, modulation of MSNs, FSIs, or both MSNs and FSIs as illustrated in A. Gray bars denote frequency bands used in the classifier in Fig 7.

After computing the aggregate signal from the spiking activity of all neurons (MUA), we examined the relationship between high frequency power in the MUA signal and the individual population firing rate of MSNs and FSIs. An example of the MSN and FSI population activity as well as simultaneous power measurements in low MUA (0.3–2 KHz) and high MUA (8–10 KHz) are shown in Fig 6A. Correlation analysis was performed between the firing rates of MSNs and FSIs against band-limited power (BLP) measurements in each frequency band within the MUA signal and at each time period. An example of the correlation analysis between one cycle of MSN firing rates and low MUA is shown (Fig 6B, from vertical lines in Fig 6A and denoted in blue circles in Fig 6C). At rest (during stochastic firing; 0–10 s), the firing rate of MSNs exhibited stronger correlation with power in lower frequencies (< 3 KHz) than higher frequencies (> 7 KHz), although this correlation was low (Fig 6C; *r = 0.1–0.2*; *small blue circles*). FSI spiking demonstrated stronger but low correlation in higher frequencies (Fig 6C; *r = 0.1–0.2*; *small red circles*). When the input drove both FSI and MSNs, the correlation between firing rate and power in both lower and higher bands increased (*r = 0.6–0.7*, *blue and red x markers*). During time periods where the input drove MSNs but not FSIs, a higher correlation was observed between MSN spiking and power in lower and higher bands (*r = 0.6–0.7*, *open blue circles*). In contrast, when inputs drove only FSIs, a correlation between FSI spiking and power in higher bands was observed (*r = 0.6–0.7*, *open red triangles*). When one population was modulated but not the other, correlations between firing rates of the opposite population and power was low for all frequency bands (*r < 0.2*, *open red circles and blue triangles*). In other words, lower MUA frequency bands correlated with a) MSN firing rates when the input drove MSN spiking only and b) MSN and FSI firing rates when the input drove MSN and FSI spiking. In contrast, higher MUA frequency bands correlated with a) FSI firing rates when the input drove FSI spiking, b) MSN firing rates when the input drove MSN spiking, and c) MSN and FSI firing rates when both populations were simultaneously responsive. The shift between low and high MUA tracking different neuronal populations appeared to exist between 3-6KHz (Fig 6C).

Taken together, low and high MUA frequency bands tracked MSN activity when MSNs were being actively driven. Interestingly, only high MUA frequency bands could track FSI spiking when FSIs were being actively driven. Correlations were higher when both populations were being driven together, although this is difficult to evaluate under experimental conditions. For example, while it is possible that a single input may simultaneously drive both MSNs and FSIs, a *continuous*, simultaneous train of inputs (similar to the simulation presented in this work) will lead to interneurons directly inhibiting MSNs, blurring the ability to evaluate this observation. There was no correlation between power in the MUA signal and the firing rate of the population not being modulated. In summary, we observed that the low MUA power was sensitive to changes in the MSN population, while high MUA power was sensitive to changes in both the MSN and FSI population.

**Fig 7:**
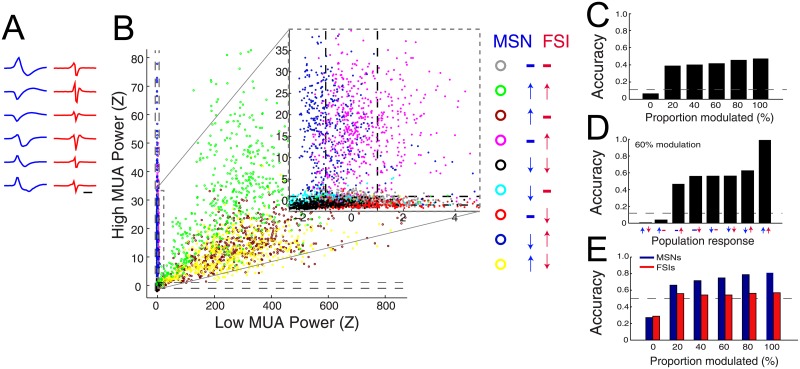
In the absence of well-isolated spikes, the spectral signature of MUA is a poor predictor of evoked neuronal ensemble dynamics. A) Representative MSN (blue) and FSI (red) spike waveforms used in simulation. Time scale = 1ms. B) Results of simulations of high frequency activity derived from spiking. Data is from simulation where 60% of neurons were modulated. Each data point represents the low and high frequency power during 10ms time windows in the simulation. Colored data points represent time bins where the instantaneous firing rate of certain populations deviated from baseline. For each color, the direction of simulated spiking activity for MSNs and FSIs is shown in the legend on the right panel. For example, green dots represent 10ms time bins where both MSNs and FSIs increased in spiking activity. Black dotted lines represent the significance threshold for determining if low or high MUA reached significance (>2SD) during a time window. The gray dotted insert shows a detailed version of data on the left of the figure. C-E) Classifier results. C) Overall accuracy for correctly predicting population activity as a function of the proportion of neurons whose activity was modulated during the simulation. Dotted line denotes chance level (8-way classifier). D) Relationship of the accuracy of detecting each type of firing rate deviation on the overall accuracy of technique. Data is shown for simulation where the input drives 60% of neurons. E) Accuracy of predicting MSN dynamics with low MUA and the accuracy of predicting FSI dynamics with high MUA. Data shown as a function of the proportion of neurons modulated during simulation.

Given the correlations observed, we next explored how well the power in low (0.3-2KHz) and high (8-10KHz) MUA frequency bands can predict the behavior of each population of neurons. The spike waveform of six representative MSNs and FSIs that were used in the simulation are shown in Fig 7A. Fig 7B plots the results of the simulation when 60% of neurons were modulated (as an illustrative example). Each data point on the scatter plot represents 10ms of time and the color of that data point represents the instantaneous population activity of MSNs and FSIs during that time compared to resting conditions. Points of gray color denote no difference of MSN or FSI population firing when compared to rest, while other colors represent significant deviations from rest. Dashed black lines in both directions represent two standard deviations from resting high frequency power. In general, an increase and decrease in MSN spiking resulted in a rightward and leftward shift in low MUA (0.3-2KHz) power, respectively, while an increase and decrease in FSI spiking resulted in an upward and downward shift of high MUA (8-10KHz) power. However, it is evident that it is extremely difficult to accurately categorize FSI spiking when MSN population spiking also increases at the same time (Fig 7B, insert).

Using these two features (low and high MUA), for each time point that deviated from rest, we calculated the accuracy of high frequency activity to predict changes in the instantaneous population firing of MSNs and FSIs. Overall, accuracy levels ranged from 6.8 to 54.5% when 0% and 100% of neurons were responsive to input (Fig 7C). As eight possibilities exist regarding the change in firing rates of MSNs and FSIs (see Fig 7D), assignment accuracy was compared to a chance assignment accuracy of 0.125. To illustrate the accuracy of the individual firing rate patterns included in the comprehensive analysis from Fig 7C, a breakdown of the simulation when 60% of neurons are driven is shown as an illustrative example in Fig 7E. In this example, the accuracy of correctly predicting time periods when MSNs increased activity and FSIs did not were below chance level, while time periods when both populations increased exceeded 90% (Fig 7D; *8-way classifier*, *12.5% chance level*). For the 2-way classifier (when either low MUA is predicting MSN patterns or high MUA is predicting FSI patterns), the ability to correctly predict the dynamics of FSIs slightly exceeded chance level (51.1% to 56.2%), while MSN prediction varied from 68.8 to 80.3% for 20% and 100% responsiveness, respectively (Fig 7F; *2-way classifier*, *50% chance level*). Note that this 2-way classifier evaluated the accuracy of predicting one subtype population activity dynamics while the other population increased, decreased, or did not change.

**Fig 8:**
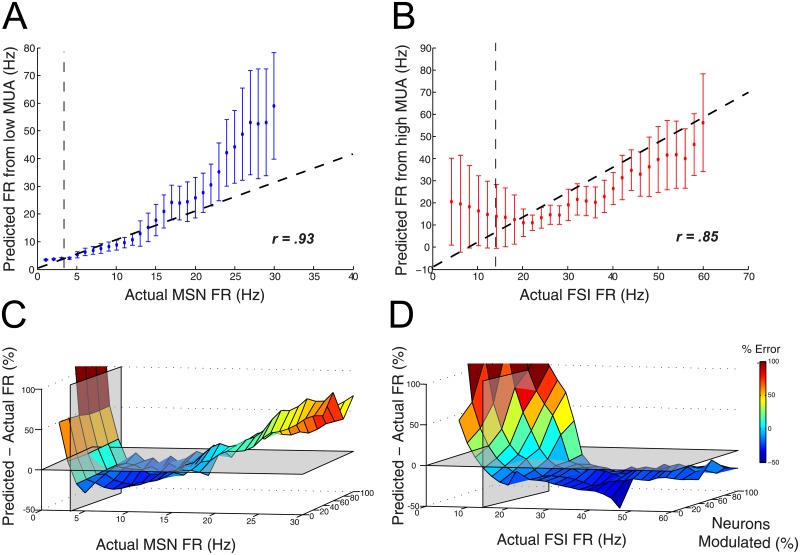
Accuracy of MUA predicting evoked population dynamics depends on population parameters. A-B) Comparison of actual and predicted population firing rate for A) MSNs from low MUA (0.3–2 KHz) and B) FSIs from high MUA (8–10 KHz). Data is from simulation where 60% of neurons were modulated. Dotted vertical line denotes mean population firing rate. Dotted diagonal line represents unity line. Error bars denote S.D. Correlation value is from linear best-fit line. Note that at high population firing rates MSNs are not predicted well from low MUA, and at low population firing rates FSIs are not predicted well from high MUA. C-D) Surface plots of prediction error for C) MSNs and D) FSIs as a function of actual population firing rate and the percent of population responsive to input. Transparent planes represent mean firing rate of population (vertical plane) and 0% prediction error (horizontal plane).

Finally, we examined the ability of MUA to predict population activity dynamics on a continuous scale as opposed to a discrete classifier. Using low MUA to predict MSN dynamics and high MUA to predict FSI dynamics, we computed a predicted population firing rate (see Methods) for each time point and compared that to the actual population firing rate for each type of neuron. As a representative example, when 60% of neurons were responsive to the input, low (0.3-2KHz) MUA closely predicted MSN population firing rates (Fig 8A, *r = 0.93*, *p < .001*, *Student’s t-statistic*, *df = 28*). Qualitatively, this was true for both at low and high firing rates, although this correspondence dropped off at higher population firing rates. Similarly, overall MUA closely predicted FSI activity (Fig 8B; *r = 0.85*, *p < .001*, *df = 28*). In contrast to MSNs, qualitatively high MUA predicted FSI spiking well at high firing rates but poorly at low firing rates. When examining these relationships as a function of firing rate and percent of neurons responsive to input, it is evident that firing rate changes had larger effects on predictability than the percent of modulated neurons (Fig 8C and 8D). At firing rates below the mean, MUA poorly predicted ensemble spiking for both populations, with >100% error below the mean firing rate of each population. For MSNs, firing rates between 3.5 to 23.4Hz yielded a <25% error in low MUA predicting MSN population spiking. For FSIs, firing rates between 10 and 35Hz yielded a <25% error in high MUA predicting FSI spiking. It is interesting to note that, for all datasets, the percent of neurons which were responsive to input did not significantly change the ability for MUA to predict population dynamics.

## Discussion

In this study we tested the efficacy of high frequency activity in extracellular recordings to predict the spiking activity of specific neuronal subtypes. We demonstrate the following:

1. in the presence of a well-isolated spike, high frequency activity in the upper LFP and MUA range accurately detected and characterized the spiking of neuronal subtypes based on the action potential’s spectral signature, where spikes with broad action potentials exhibited more power in frequencies below 2KHz compared to spikes with narrow action potentials.
2. Neuronal subtypes could be categorized based on features of the high frequency activity at the single trial level with 80% accuracy.
3. Simulations of neuronal populations demonstrated that low MUA tracked MSN activity, while high MUA tracked both MSN and FSI populations. When predicting the direction and subtype class of *both* types of neurons, MUA predicted the correct pattern with 40–50% accuracy; above the chance level but not high enough for practical implementation. Individually, low MUA predicted the MSN pattern with 60–80% accuracy, raising the possibility of practical use. However, this accuracy decreased significantly when MSNs were inhibited. On the other hand, high MUA predicted the FSI pattern with accuracy around chance and the accuracy decreased further when FSIs were inhibited.

We conclude that in general MUA activity is a poor predictor of specific population activity and that only during specific conditions may MUA potentially have practical applications. Regardless of the condition, spectral components of spikes within each population must be known, but even then the separation of populations within MUA must be performed with caution.

### From spiking to high frequency activity

Converging evidence suggests that components of spiking are represented in extracellular recordings at frequencies >100Hz. In this work, the probability that a spike elicited significant increases in high frequency activity was <50% for frequencies <100Hz, >50% for frequencies >100Hz, and maximum (at 75%) at 1KHz. This data suggests that LFP power <100Hz (and especially <30Hz) does not provide a good proxy for spiking activity, whereas high frequency activity >100Hz at least partially represents spiking activity in the dorsal striatum. This data is consistent with previous work reporting a strong correlation between spiking and LFP activity > 100Hz in primate visual cortex [13, 14, 17, 18], rodent hippocampus [11, 19], and human auditory cortex [20, 21]. The variety of species and structures exhibiting this correspondence supports the notion that this relationship may be ubiquitous.

### Spectral signatures of neuronal subtypes

Spikes from different neuronal classes have extracellular spectral signatures than can sometimes by accurately categorized using these features. In this work, action potentials with broader spike widths identified as MSNs exhibited higher power in lower MUA (0.3-2KHz), whereas FSIs with narrower spike widths exhibited greater power in higher MUA (8-10KHz). Furthermore, the power in low and high frequency components were able to differentiate neuronal subtypes with 80% accuracy on a trial-to-trial basis. The observation that spiking is represented in the LFP frequency range (<250Hz) [17, 22] has motivated groups to examine the “despiked” LFP [22, 23]. Indeed, a significantly larger power decrement at frequencies >150Hz was observed following hippocampal pyramidal cell “despiking” compared to when interneurons were removed [11], supporting our observation as pyramidal cell spikes have broader spike widths compared to fast-spiking interneurons [24]. Unfortunately, analysis in this prior study was confined to frequencies below 1KHz [11], above which we would predict interneuron “despiking” to result in a larger power decrement compared to pyramidal cell removal. In summary, spectral signatures appear to be able to in principle differentiate neuronal classes based on their spike waveform.

### Tracking neuronal populations with high frequency activity

Because of the ambiguous neuronal source of not well isolated action potentials, it is difficult to examine the effect of background spiking on MUA [11]. For these reasons, we modeled this ‘background’ neuronal activity and assessed the degree to which MUA predicted population spiking. Our results demonstrate that utilizing background spiking activity to track moment-to-moment population firing rates depends on the spike waveform, distributions of neuronal classes, and the strength of responses. Accuracy was highly dependent on the *direction* of the change in the activity (increase or decrease) and *type* of neuron that was modulated and was less dependent on the *proportion* of neurons that were modulated. High MUA predicted FSI activity less accurately than low MUA predicted MSN activity. A likely explanation is that the MSN spike waveform consists of both high and low frequency components; an initial sharp rise during the depolarization phase followed by a slower hyperpolarization and overshoot. Thus when MSNs increased their firing rate it was possible to detect their increased activity, but as MSN spiking leaks into higher frequencies, FSI changes, if present, were not discernable (Fig 7C and 7E). In contrast, because the FSI spike waveforms consist primarily of sharp high frequency deflections, changes in their spiking activity were evident only in the high MUA band.

Although it is tempting to conclude that low MUA may be used to predict MSN or other populations with broad spikes, one must proceed with caution. First, when using low and high MUA to predict both populations’ activity, accuracy was 40–50% regardless of the proportion of neurons that were modulated. Although this was well above chance level (12.5%), it is important to note that *above chance* does not necessarily mean above a threshold that becomes practical to implement. With <50% discriminability, this technique cannot be used reliably to predict both populations’ activity. However, it may be possible to use low MUA to predict MSN activity. During times when the input drove MSN spiking, low MUA power predicted the direction of MSN spiking changes with an accuracy exceeding 70% (Fig 7F), well above chance (50%) and above the threshold to support practicality. This high level of discriminability was evident regardless of the proportion of MSNs modulated. When examining this in more detail, however, the ability to predict MSN activity decreases sharply at firing rates below the mean as well as at very high firing rates (Fig 8C), thus significantly limiting its practical use. Often physiological events involve periods of excitation paired with inhibition; therefore, it may not be feasible for low MUA to accurately monitor MSN activity during many physiological events.

### Limitations of estimating population activity from MUA

Although the spike waveforms and firing rates in the simulation were derived from experimental recordings and neuronal distributions were comparable to previous reports [16, 25], several differences between these simulations and experimental recordings may confound the ability to use the MUA to track neuronal populations *in vivo*. First, in our simulations we did not vary the distance from the neuron’s spike initiation zone to the recording site. *In vivo*, the heterogeneous distances from each neuron to the recording electrode would result in a distribution of spike amplitudes and waveforms. Presumably this filtering of action potential waveforms may lower the overall frequency content of each spike, potentially blurring the divide between frequency bands. Second, neuronal synchronization has been shown to influence high frequency activity [18] but was not varied in this simulation and likely will affect the ability to track populations at various synchronization levels. Third, it is important to note that even in the idealized situation of inserting the average spike waveform to represent single action potentials, it was difficult to use MUA to separate and predict the temporal dynamics of neuronal populations. Fourth, these simulations assumed that activity >300Hz is comprised solely of action potentials. While the notion that MUA primarily reflects spiking activity is generally accepted, fast components of excitatory and inhibitory post-synaptic potentials may be partially represented in these frequencies. It should be noted, however, that the simplicity of the simulation reported here strengthens the argument that it is difficult to predict population activity from different neuronal classes without prior comprehensive understanding of the underlying neuronal distributions and population activity. Thus although a more complex simulation should be performed that takes these factors into account, it is likely that adding complexity to the origin of MUA by incorporating fast EPSP/IPSPs into the signal, lowering the signal-to-noise ratio by using non-averaged spike waveforms, and simulating spikes generated from heterogeneous distances from the microelectrode together will further lessen the ability to predict population spiking from specific frequency bands. Together, these important considerations highlight the fact that only population spiking from certain neuron subtypes were predicted from background multi-unit activity and this applicability likely diminishes for *in vivo* recordings. In conclusion, we suggest that MUA cannot be used to estimate population spiking without prior knowledge of spectral components of spikes within each neuronal class.

On the other hand, several considerations should be noted that may increase the ability for the multiunit signal to track population dynamics. First, recordings in this report were conducted on awake, freely moving mice. Presumably, in a behavioral task where neurons modulate their spiking activity in a more homogeneous manner, population activity may be better extracted from multiunit activity. Second, the ability to predict population activity with the multiunit signal may vary between regions. MSNs receive multiple synaptic inputs yet have low overall firing rates. This input/output discrepancy is less in other regions, such as the hippocampus. Furthermore, in other regions, the overall firing rate of the principal neuron is much higher than MSNs (i.e. Purkinje cells in the cerebellum). Therefore, the ability to predict population activity from multiunit signal may be higher in regions whose neurons’ firing rate is higher. As such, this work should be repeated in other regions before these findings can be generalized.

## Materials and Methods

Experiments were performed in accordance with guidelines set by and with specific written approval from the Institutional Animal Care and Use Committee of Albert Einstein College of Medicine. Experiments were conducted on 8–10 week old C57/Bl6 mice of either sex. 17 total mice were used for this study. Mice were allowed at least four days to recover from surgery before recording. The main findings of this manuscript did not differ across animals or recording days. Following the completion of experiments, animals were sacrificed by administering the anesthetic isoflurane, perfused with 4% paraformaldehyde. Administration of isoflurane was performed in order to alleviate the animals’ suffering. Brains were recovered for histological verification of recording sites.

### Electrodes and implantation

Custom-made drivable 8-microwire (Tungsten, 50 μm, AM Systems) arrays [26] were implanted into the dorsolateral striatum (ML 2.5 mm; AP 0.5 mm; DV 1.5 mm). Signals were referenced to an uninsulated wire in the array. In the absence of well-isolated spikes, arrays were advanced at 75 or 150 μm intervals for a maximum of 150 μm/week. In each animal, electrodes were advanced a maximum of 1.5 mm such that the recordings were confined to the dorsal aspect of the striatum. Electrode positions were confirmed by lesioning (60 μA, through each electrode for 30 s) and postmortem histology.

### Recordings

Signals were amplified 5000x with a headstage (Tucker-Davis Technologies) and a homemade amplifier (150 Hz—10 kHz, RC bandpass) and digitized at 20 kHz with a National Instruments card (PCI-MIO-16XE). Signals were acquired using custom-written software in Labview. To analyze single unit activity, signals were wavelet filtered [27] in MATLAB (Mathworks, Natick, MA) and sorted offline using principle component analysis (Offline Sorter, Plexon, Dallas, TX). Units exceeding 4x signal SD were included in all subsequent analyses. Waveform shapes were quantified in MATLAB. Brief waveform signals (putative interneurons) were defined as those units which had positive deflections with full width at half maximum (FWHM or ‘spike width’) < 200 μs. Long duration waveform signals (putative medium spiny neurons) were defined as those units which had positive deflections with FWHM > 200 μs and firing rates of < 2 Hz [16, 25].

### Frequency analysis

To represent wideband (0.3 Hz– 10 KHz) recordings in frequency space, we utilized two approaches. When examining the frequency content of spike waveforms, the data segment of interest was extracted (10 ms before and after the spike) and transformed using a fast-fourier algorithm (MATLAB) with 2048 frequency bands from 0.3 Hz– 10 KHz. To account for differences in spike amplitude that may reflect recording sampling biases rather than physiological differences, the output of each frequency transform was normalized to the area under the frequency decomposition curve. Power in the 4500-5000Hz band has been removed due to environmental noise peaking at this frequency band.

For longer periods of data, we calculated band-limited power (BLP) for discrete frequency bands by calculating the square of the bandpass-filtered data (zero-phase, 5-pole Butterworth filter, MATLAB). For all analyses, BLP in typical EEG bands <100 Hz (theta 4–8 Hz, alpha 8–12 Hz, beta 12–25 Hz, gamma 30–100 Hz) as well high frequency bands (100 Hz wide bands >100 Hz; i.e., 100–200 Hz, 200–300 Hz, 900–1000 Hz, …9900–10000 Hz) were computed. For each frequency band, we calculated the correlation coefficient between the spike waveform attributes from all neurons and either the power in the frequency decomposition (for short segment data) or the total BLP over five minutes (for continuous data). An example of the resulting scatterplots for 300–400 Hz is shown for visualization purposes in Fig 1. Subsequently, the resultant r-value for each frequency band is plotted as a function of the logarithm of frequency. Statistical tests for all correlations were carried out by transforming the correlation value r to a t-statistic with N number of samples and N-2 degrees of freedom: $t=r(\surd \left(N-2\right)/\surd \left(1-r^{2}\right).$

### Detecting spikes with high frequency power

To evaluate the ability of high frequency activity to detect spike timing, we first calculated the z-score of the BLP in each frequency band. For each spike, the mean BLP during the period of spiking (+/- 1ms) was calculated. If the mean BLP exceeded 2 SD during this time, it was considered a detection event (true positive). For all time periods not including a spike, if the BLP was below threshold it was considered a true negative. The sensitivity (true positive rate) and specificity (true negative rate) of BLP to detect spiking were then calculated. Varying the BLP threshold did not significantly alter the sensitivity or specificity results. This analysis was performed for each spike. Sensitivity and specificity were plotted as a function of frequency for all neurons.

### Single spike classifier

To determine the accuracy of high frequency activity in detecting and classifying spikes, we implemented a support vector machine (SVM) via the SVM train-and-classify routines in MATLAB [28, 29]. Briefly, an SVM finds a hyperplane that separates all data points into two classes with the largest margin. The margin refers to the maximal distance orthogonal to the hyperplane containing no data points. Support vectors refer to data points closest the separating hyperplane. Thus, for a set of training data consisting of data points x_i_ and their groups y_i_ (in this case MSNs or FSIs), a hyperplane can be defined as:

<w,x>+ b = 0

Where w ∑ R^d^ and <w,x> is the dot product of w and x. In this context, the best hyperplane is defined as:

$$y_{i}\left(<w,x_{i}>+b\right)\geq 1$$

and support vectors are those xi on the boundary:

$$y_{i}\left(<w,x_{i}>+b\right)=1$$

After the best hyperplane is defined, the class that a vector z belongs to can be represented by:

class(z)=sign(<w,z>)+b.

First, multiunit power (>300 Hz) measurements that exceeded 1 SD were extracted (+/-25 ms) and a frequency transformation was performed for each data segment and normalized to the area under the curve. Next, for each data segment, power in the lower (500-600Hz) and the higher (3000-3500Hz) bands were computed and used as features in a binary classifier. These frequency bands were chosen as they exhibited the largest power separation between MSNs and FSIs (Fig 3B). For all analysis, the classifier was trained on 25% of cells selected at random, while classification was performed on the remaining 75% of cells. Finally, the accuracy of neurons in the classification dataset was calculated for each cell based on single trial data. For each neuron, misses (times of no BLP increase during known spiking) were incorporated into accuracy measurements.

### Modeling multiunit activity

As it is unclear how different neuronal populations contribute to activity in the multiunit frequency range, we modeled a dorsal striatum population with known spiking parameters to examine how changes in population firing rates affect multiunit activity. 20,000 cells were used in this simulation, with a distribution of 95: 5% MSNs: FSIs. This distribution represents the actual distribution of these two neuronal populations in the striatum [30]. For each cell, we created a set of Poisson distributed time points in order to model the spiking of a neuron. In these simulations, this distribution was used to model spike *timing*, while the average action potential waveform of spikes recorded experimentally were inserted in the time series in order to perform frequency analysis on the dataset, as is described below. Mean and standard deviation of MSN and FSI spiking in the model was constrained to match those in the experimental dataset (2.4Hz +/- 3.6 SD for MSNs; 16.1Hz +/- 10.2 SD for FSIs). To model the effect of activation or suppression of a specific subset of neurons, we modulated the firing rate of none, one, or both of the subtype populations. The spike timing distribution for each cell was convolved with a 0.5 Hz sine wave to modulate the proportion of spiking cells. Time periods of modulation of specific subtypes can be found in Fig 5A. The timing of the conditions were as follows: spontaneous firing (no modulation; 0–10 s); MSN modulation (10–20 s), FSI modulation (20–30 s), modulation of MSNs and FSIs such that changes are negatively correlated (30–40 s), and modulation of MSNs and FSIs such that changes are simultaneous and positively correlated (40–50 s). Simulations were run such that during time periods of neuronal modulation, 0, 20, 40, 60, 80, or 100% of the neurons in that population were responsive to the sinusoidal input.

The raster plot for a subset of MSNs and FSIs as well as the binned population firing of all cells are displayed in Fig 5B. For each of the 20,000 neurons, a spike waveform was selected at random from the recorded dataset depending on the subtype. This spike waveform was inserted into the simulated data each time that spike fired. This procedure was repeated for all neurons in the simulation. Non-neuronal noise was incorporated into the simulation by utilizing 50 s of data from recordings from a dead mouse (recorded following the administration of 10% isoflurane for 10 minutes). Noise levels in the simulation were adjusted to match the ratio from pre-death to post-death wideband activity in the experimental recording. The majority of this noise consisted of 60 Hz and did not significantly affect accuracy measurements. Finally, spike waveform data from each neuron was summed to calculate the MSN and FSI contribution to high frequency activity and noise was added as described below. Simulations were repeated five times and averaged to account for possible variations between runs.

### Analysis of simulated MUA

To compare spiking activity to activity in each frequency band, data was band-passed (5-pole, Butterworth zero-phase filter, MATLAB) and squared to compute the band-limited power. To determine the degree to which different frequencies ‘track’ population firing rate changes in specific cell types, a Pearson’s correlation coefficient was computed between the BLP of the frequency of interest and the population firing during time periods of each 10 s modulation condition.

Then, a classifier was implemented in order to calculate the accuracy of BLP to detect and track population ensembles. Instantaneous firing rates for both cell types were calculated for each 10 ms time bin for the duration of the simulation. The mean BLP in each 10 ms time bin for lower frequencies within the MUA range (0.3–2 kHz, termed ‘low MUA’) and higher frequencies (8–10 kHz, ‘high MUA’) were calculated. These frequency bands were chosen as they demonstrated clear differences in their ability to track the different populations (Fig 6C). For each time point where the sine wave input deviated from resting values (0–10 s), we categorized BLP changes in both frequency bands as exhibiting no difference or deviating from baseline changes as determined by >2 SD from resting time periods. Finally, for each time point, changes in low and high MUA were compared to population firing rates of each cell type. Two different classifiers were implemented. We first examined the ability for low and high MUA to correctly predict both the pattern of MSN and FSI firing rates. In this analysis, an 8-way classifier (eight possible changes between both populations; see Fig 7D) was used such that “chance accuracy” was 1/8 or 12.5%. The second classifier examined the overall accuracy for low MUA to predict MSN firing rates and high MUA to predict FSI firing rates individually. Here, when analyzing low MUA, only data points where the MSN population firing rate deviated from baseline were included. Importantly, data points where FSI populations were modulated simultaneously with MSNs were included in this classifier. In this case, a chance level of 50% accuracy (predicting that MSNs either increased or decreased firing rates) was implemented. The same analysis was performed for high MUA to predict FSI activity.

Finally, we evaluate the predictability of MUA on population firing rates on a continuous scale instead of a discrete classifier. To do so, we generated predicted firing rate traces of MSNs from high MUA and predicted traces of FSIs from low MUA. After converting each MUA trace to z-scores, we 1) scaled the trace by the standard deviation of the resting period (0–10 s) and 2) shifted the trace by the mean firing rate of this time period. Next, we computed the percent error from the predicted and actual firing rate traces at each time point for each population. For example, the actual MSN firing rate trace was compared to the predicted MSN trace derived from low MUA. This analysis was performed for all simulations and for each population. Finally, surface plots (one for MSNs and low MUA and one for FSIs and high MUA) were created to visualize the percent error from each population while varying the total population firing rate and the percent of modulated neurons.

## Supporting Information

S1 Fig Modeling the spiking contribution to high frequency activity. A) Determining the effect of spike width on spectral power while holding firing rate and amplitude constant. Top panel: Examples of modulating the width of one spike. Middle panel: Relationship of width to total power in 300-400Hz band over five minutes of recording. Bottom panel: Comparison of spike width to all frequencies while constraining amplitude and firing rate. B-C) Determining the effect of modulating spike B) amplitude and C) firing rate on spectral power over the five minute recording.
(EPS)
